# Regarding: ‘risk factors of venous thromboembolism in ICU patients: a systematic review and meta-analysis’

**DOI:** 10.1080/07853890.2026.2638850

**Published:** 2026-03-18

**Authors:** Junming Li, Yixun Huang

**Affiliations:** Department of Emergency Medicine, Linping Campus, The Second Affiliated Hospital of Zhejiang University School of Medicine, Hangzhou, China

To the Editor

We read with interest the systematic review and meta-analysis by Yang and colleagues on factors reported to be associated with venous thromboembolism (VTE) among patients admitted to the intensive care unit (ICU) [1]. Based on observational studies, the authors summarized associations between ICU-related VTE and several patient- and treatment-related characteristics, including central venous catheterization, invasive mechanical ventilation, older age, prolonged ICU length of stay, malignancy, elevated D-dimer levels, and a prior history of VTE. This review brings together findings that have been described across multiple cohorts of critically ill patients. We would like to comment on several methodological aspects that may be relevant when interpreting these results.

Reported VTE incidence varied substantially across the included studies, which may partly be related to differences in diagnostic approaches and thromboprophylaxis practices. In some cohorts, imaging was performed mainly in response to clinical suspicion, whereas others applied routine ultrasound screening. Routine screening is known to increase detection of asymptomatic deep vein thrombosis; however, higher detection rates do not necessarily correspond to differences in patient-relevant outcomes. In a randomized clinical trial conducted in ICU patients with severe COVID-19, routine ultrasound screening identified more thrombotic events than usual care, while no clear differences in clinical outcomes were observed [2]. Although this study focused on a specific patient population, it suggests that variation in surveillance intensity can influence reported event rates and should be considered when comparing results across observational studies.

Several characteristics identified in the meta-analysis, such as invasive mechanical ventilation, central venous catheterization, and ICU length of stay, change over the course of hospitalization. Analyses that treat these variables as fixed baseline characteristics may be affected by time-related analytical issues that arise in longitudinal ICU research [3]. Approaches that account for changes over time, including time-to-event analyses with time-varying covariates or landmark methods, may help to better capture the evolving nature of ICU care.

The interpretation of D-dimer measurements and risk assessment models in ICU populations also requires caution. Although higher D-dimer levels were frequently reported in patients with VTE, thresholds differed across studies and laboratory methods were not uniform. In addition, ICU-specific risk assessment models have been evaluated in selected cohorts, but evidence from external validation studies remains limited [4]. Tools developed for non-ICU settings, such as the Caprini score, may not fully reflect the clinical context of critical illness, given differences in disease severity and physiological instability [5]. Further work focusing on models tailored to ICU populations may help clarify their potential role in clinical decision-making.

In conclusion, the meta-analysis by Yang et al. summarizes factors that have been reported to be associated with VTE in critically ill patients [1]. Consideration of diagnostic strategies, attention to time-varying clinical characteristics, and careful interpretation of risk assessment tools may assist readers in applying these findings to future research and clinical practice.

## Data Availability

Data sharing is not applicable to this article as no data were created or analysed in this research.

## References

[1] Yang X, Zhou X, Qiu Y. Risk factors of venous thromboembolism in ICU patients: a systematic review and meta-analysis. Ann Med. 2026;58(1):2614222. doi: 10.1080/07853890.2026.2614222. 41527456 PMC12802517

[2] De La Puente CEM, Flota Ruiz D, Sánchez Besalduch L, et al. Systematic ultrasound screening for lower extremity deep vein thrombosis in ICU patients with severe COVID-19: a randomized clinical trial. J Intensive Care Med. 2025;40(7):739–748. doi: 10.1177/08850666251313774. 39838947

[3] Vail EA, Gershengorn HB, Wunsch H, et al. Attention to immortal time bias in critical care research. Am J Respir Crit Care Med. 2021;203(10):1222–1229. doi: 10.1164/rccm.202008-3238CP. 33761299

[4] Rivrud SCS, Heijkoop ERH, Hulshof L, et al. Predicting the risk of venous thromboembolism in critically ill patients (PROVE-IT): a model development and validation study. J Thromb Haemost. 2025;23(10):3272–3285. doi: 10.1016/j.jtha.2025.06.026. 40617504

[5] Caprini JA. Individual risk assessment is the best strategy for thromboembolic prophylaxis. Dis Mon. 2010;56(10):552–559. doi: 10.1016/j.disamonth.2010.06.007. 20971324

